# 

**Published:** 1887-02-05

**Authors:** 


### The Hospital.

An Institution, Family, and Parochial Journal of Hospitals, Asylums, and all Agencies for the Care of the Sick, Criticism and News.

SATURDAY, FEBRUARY 5TH, 1887.

### The Literary Prize.

A PRIZE of **Two Guineas** will be given every month for the best story or incident based upon hospital, or asylum, or student life, or any incident connected with the professions of medicine or nursing.

### The Quilter Prizes.

### HOSPITAL HOUSEKEEPING AND MANAGEMENT.

We offer two prizes of Ten Guineas and Five Guineas respectively for the two best reports on the Expenditure of any single hospital, such reports to give all the details concerning the cost of food and stimulants, brought out in detail in the table on page ii. of the Special Supplement we publish to-day, together with a critical examination of the various recommendations contained in the report of the Special Committee of the Newcastle Infirmary, and the addition of the figures to the following heads of expenditure. Hospital secretaries may procure a printed form of return, containing all the particulars to be filled up by competitors, on application to the Editor.

1. *Domestic Expenses.*—Soap and Soda; Laundry, Repairs, and Washing; Water Rate; Coals, Coke, and Wood; Gas and Oil; Bedding and Linen; Furniture, Earthenware, and Hardware; Uniform and Livery, for *(a)* Porters, *(b)* Sisters and Nurses.
2. *Surgery and Dispensary.*—Drugs; Surgical Dressings and Bandages; Instruments, Artificial Limbs and Trusses; Mineral Waters; Dispensary Sundries.
3. *Salaries and Wages.*—*(a)* Management, including Secretary, Superintendent, Clerks, and Collectors (including commission); *(b)* Nursing Department, Matron or Lady Superintendent, Superintendents of Nursing, Sisters, Nurses, Extra Nurses, Ward Maids and Scourers; *(c)* Maintenance of Porters and Servants (not included in *a* and *b*).
4. *Management Expenses.*—Printing and Stationery; Advertisements; Postage; Special Appeal for Funds; Annual Court and Festival; Dinner; Auditor's Fee; Banker's Interest.
5. *Rates and Insurance* (exclusive of Water and Gas rates).
6. *Pensions*, showing to which Department they belong.
7. *Repairs and alterations of Structure.*—*(a)* General, including flooring and painting; *(b)* Extraordinary including building.
8. *Miscellaneous Expenses* (*i.e.* all other items to be given in detail).

All the figures are to be those for the year 1886, and the totals must agree with the figures published in the annual report, and copy of which must be sent in with the manuscript. Competitors must enclose their name and address, must write on one side of the paper only, and give the name of the hospital they have selected. No competition should occupy more space than that represented by three pages of printed matter in The Hospital.

All competitions must be sent in not later than the 31st of March next, addressed to the Editor, The Hospital, Norfolk House, Norfolk Street, London, W.C., and it is hoped that particulars may in this way be supplied from every British Hospital.

### AMUSEMENTS WITH PROFIT.

### PRIZE COMPETITIONS.

**Quarterly Prize Competition.**
Began 22nd January; Ends 16th April.

**A Prize of Three Guineas**
will be awarded to the Competitor who shall have sent in the largest number of correct or best answers. No one will be permitted to win the Three Guinea Prize twice in any one year, but we shall award annually an additional
**Prize of Five Guineas**
to the competitor who shall have sent in the largest number of correct or best answers during the twelve months.

**No. 5. Double Acrostic.**

Three Hospitals Royal old London possesses
One of these, dear friends, you here will see.
By a worthy prior of Bermondsey founded:—
My primals and finals tell which of the three.

Deep heavy sound, so expressive of grief
We *heave* it oft, in chance may bring relief.

Down in a dell on the *Cornish coast* I lie,
Three streets, they say, my name doth signify.

Who these may lose will never find again,
Each day *same number* brings to king and swain.

"This runner doth outstrip them all," he cried—
Look keenly and the *light* will be espied.

A narrowish *arm* of the Irish sea
Dividing Carnarvon from Anglesea.

A *mountain* of note in Biblical lore,
For here the Ark rested in days of yore.

A writer whose initials are like *gas;*
This is enough; so to the next I'll pass.

A name once owned by the Apostle *Paul*,
It will, I am certain, be guessed by all.

**No. 6. "Hospital."**
Give, in prose or verse, the best definition you can of the word "Hospital." No answer may exceed fifty words.

**Answers.**
No. 1. DOUBLE ACROSTIC.

S elfis H

G eneralissim O

E ar S

O wnershi P

R abb I

G rea T

E tn A

S choo L

Correct answers have been received from Ostrich, A. M. E., Mater, K. M., H. B., Tabs, Condorcet, Ellaby, Miss Larkins, Patient, H. J. C., Paddy, Perseverance, Ellice, Mr. J. High.

FREDERICK, C.—You have two "lights" wrong.

GRETTA.—Three "lights" wrong.

MR. J. HIGH.—There is no entrance fee.

No. 2. A HOSPITAL APPEAL.

We have received several decidedly clever answers, a selection from which we hope to publish. All our competitors with three exceptions, chose verse to give expression to their APPEAL.

Best answers have been received from Patient, Gretta, Ellaby, Condorcet, Tabs, H. B., K. M., Mater, A. M. E., Ostrich, Ellice, Perseverance, Paddy, H. J. C.

Answers must be addressed to THE PRIZE COMPETITOR, THE HOSPITAL, Norfolk House, Norfolk Street, Strand, W.C., not later than Saturday next. The full name and address must in all cases be given, but a *nom de plume* may be added for publication. Only one side of the paper should be written on. The "Prize Competitor" Coupon (see advertisement pages) must be enclosed with replies.

### THE CHILDREN'S CORNER.

### OUR PASTIMES.

**Children's Quarterly Prize Competition.**

Began 1st Jan., 1887 : ends 26th Mar., 1887.

PRIZES.

FOR MOST CORRECT ANSWERS.

1st .. .. Thirty Shillings | 3rd .. .. Ten Shillings

2nd .. .. Twenty .. | 4th .. .. Five „

FOR NEATEST ANSWERS.
Five Shillings.

FOR BEST LITTLE LETTERS.
Ten Shillings.

**Sixth Competition.**

No. 33. CONUNDRUM. Why does Mr. Henry Irving's company at the Lyceum resemble Messrs. Maskelyne and Cooke's entertainment?

No. 34. DECAPITATION.

My first is a bird and he's quite absurd,
For if you behead him you'll find
An author of fame will quickly lay claim
To the part that is left behind.

No. 35. DIAMOND PUZZLE.

```
      *
    . * .
  . . * . .
* * * * * * *
  . . * . .
    . * .
      *
```

A summer vegetable; an animal and also an instrument for battering walls; what one longs to do in a confectioner's shop; an amusement; what the sun and moon do; a girl's name; the *end* of every pantomine. My verticals down the centre are the same as my middle horizontal line.

No. 36. My *first* is in Grecian,
And also in Greek,
My *next's* in Utopian
Likewise in bézique,
My *third* is in Yorick,
Likewise, too, in yore,
My *last* is in eagles,
And also in soar.
My *whole* is a great hospital,
I pray you remember,
'Tis also those personages
We see in November.

**Results of Fourth Competition.**

No. 23. CONUNDRUM. Richmond resembles "R" for two reasons; first, because it is next to Kew (Q), and secondly, because it is, like "R," in Surrey.

No. 24. HIDDEN PROVERB. This is, "A stitch in time saves nine."

No. 25. CONUNDRUM. The merchant's City address is more likely to be remembered because it is sure to be E.C. (easy).

No. 26. SQUARE WORDS. The *lights* here are

```

C L A R A

L E G A L

A G E N T

R A N G E

A L T E R

```

No. 27. CROSS IN A DIAMOND.

```

        H

      n O t

    ha  S  te

  stu   P   efy

P H Y S I C I A N

  rep   T   ile

    pl  A  in

      a L l

        S

```

*Five right*—Tim, Prospect, Alsie, Ellie, Percy, A. C. K.; *Four right*—Florrie, Skye, Joe, Scrooge, P. S., Paul Methven Mikado, Dot; *Three right*—Isobel, Bismarck, Ariadne; *Two right*—Peeler, Little Mary.

**Letters from Little Folk.**

Week by week the little batch of correspondence grows more and more interesting. Here is a funny little story about two poor sparrows:—

DEAR MR. EDITOR,—We have a sun-blind over our dining-room window, in which a pair of sparrows built their nest; but when the blind was pulled down, the nest being made on it, it of course came down too. Well, they built their nest and laid two or three eggs. But before they were hatched, the blind was pulled down again, and the nest and eggs came with it. Still they were not to be beaten, so next year they tried again, and got so far as to hatch three little ones, but they came down with the nest before they had got any feathers on at all. They tried again however; this time they succeeded, for in some mysterious way they fastened the nest to the cover of the blind, and not to the blind itself; so that when the blind was pulled down, it did not hurt the nest at all. I think it was very clever of them indeed. P. S.
40, Warwick Gardens, Kensington.

I am pleased to welcome a new little contributor, who forwards me the following interesting note:—

DEAR MR. EDITOR,—I have been thinking over what subject to write to you about, and have arrived at the conclusion that a few words concerning the Orphanage of Mercy would not be unsuitable. This Orphanage, situated in a poor part of London, namely, Kilburn, differs from others in one respect; no entrance-fee is required to place a child in this home; the only requisites are that it has tobe entirely destitute, and without a friend in the world. If I was allowed space I would tell you more about this home and its everyday life. To whom does this Orphanage belong? you ask, dear Mr. Editor. It belongs to the greatest, or one of the greatest blessings we have, the "Church Extension Association." If you feel interested in what I have just written, and desire further information, write to Miss Thomas, 27, Kilburn Park Road, London. I really must close this letter now. So now, I am, yours truly,
Eaton, Chester. PEELER.

Isobel must, I am sure, have quite a little aviary; here is a letter from her about her pretty pets:—

DEAR MR. EDITOR,—I think perhaps you may like to hear something about our pet birds. We have five—a linnet, canary, goldfinch, red-pole, and dove. The linnet is very tame, he will eat out of my mouth, off my finger, and if I put some seed on the tip of my nose he will take it. He also pecks my finger if I put it in the bars. The linnet and canary built a nest together last year; we fastened a round basket in their cage, and gave them some tow, cotton-wool, moss, and string, with which they made a beautiful nest; and though the canary laid six eggs (I think), she ate them all up, even the shells, which vexed us very much, as we had hoped to rear some young birds. Yours truly,
Vicars' Court, Lincoln. ISOBEL.

**Letter Prize.**

One mark each: Florrie, Skye, Peeler, Isobel, Tim, Scrooge, P. S., Mikado, H. G. B., and Little Mary.

**Notices to Correspondents.**

SKYE.—Many thanks for the charming little card you have so kindly painted for me. I have placed it in my album.

H. G. B.—May I offer you my congratulations upon the attainment of your ninth birthday, and wish you many happy returns of the day?

TIM.—An excellent little letter; I was sure you *could* write.

FLORRIE.—Thank you kindly for the little puzzle. It is a very good one.

SCROOGE.—A very nice little letter. I should much like to hear something as to what little studies you prefer at school.

MIKADO.—What a wonderful duck that was! What became of him? Thanks for your conundrums.

Answers, having the actual name and address (to which a pseudonym may be added for publication), must have "The Children's" Coupon attached (see advertisement pages), and be addressed to the "PUZZLE EDITOR," THE HOSPITAL, Norfolk House, Norfolk Street, Strand, W.C., not later than Thursday next.

### NOTICE TO CORRESPONDENTS.

All correspondence must be written on one side of the paper only.

We cannot undertake to return MSS. not used.

**Communications**, whether intended for insertion or for private information, must be authenticated by the names and addresses of the writers, not necessarily for publication.

**Local papers** containing reports or news paragraphs **should be marked.**

It is especially requested that early intelligence of **local events of interest**, or which it may be thought desirable to bring under notice, may be sent direct to the Editor.

Communications respecting editorial matters should be addressed to the Editor, Norfolk House, Norfolk Street, Strand, London, W.C.

### BUSINESS COMMUNICATIONS AND ADVERTISEMENTS.

All communications concerning business matters, non-delivery of THE HOSPITAL, etc., should be addressed to the Manager, at the Publishing Offices, 30 and 32, Sardinia Street, Lincoln's Inn Fields, London, W.C.

**Cheap Advertisements** of the "Wanted" class, including those for situations, of Twenty-four Words or under, are charged *1s.* an insertion, or three insertions for *2s. 6d.*, but must be paid for in advance. Each additional line of Eight Words is *4d.*

**Employers requiring assistants** are charged for one insertion of Twenty-four Words or under, *2s. 6d.*, or *6s.* for three insertions. Each additional line of Eight Words is *1s.*

**Advertisements** for insertion in the weekly issue of THE HOSPITAL **must be delivered** at the Publishing Offices **not later than 11 a.m. on Wednesdays.**

